# Activation of P2X_7_-mediated apoptosis Inhibits DMBA/TPA-induced formation of skin papillomas and cancer in mice

**DOI:** 10.1186/1471-2407-9-114

**Published:** 2009-04-20

**Authors:** Wen Fu, Tom McCormick, Xiaoping Qi, Liping Luo, Lingyin Zhou, Xin Li, Bing-Cheng Wang, Heidi E Gibbons, Fadi W Abdul-Karim, George I Gorodeski

**Affiliations:** 1 Department of Reproductive Biology, Case Western Reserve University, Cleveland, Ohio, USA; 2 Department of Dermatology, Case Western Reserve University, Cleveland, Ohio, USA; 3 Department of Pharmacology, Case Western Reserve University, Cleveland, Ohio, USA; 4 Department of Physiology and Biophysics, Case Western Reserve University, Cleveland, Ohio, USA; 5 The Biostatistics office at the Department of Reproductive Biology, Case Western Reserve University, Cleveland, Ohio, USA; 6 Department of Pathology, Case Western Reserve University, Cleveland, Ohio, USA; 7 Department of Oncology and the Comprehensive Cancer Center, Case Western Reserve University, Cleveland, Ohio, USA

## Abstract

**Background:**

The study tested the hypothesis that apoptosis can prevent and control growth of neoplastic cells. Previous studies in-vitro have shown that the pro-apoptotic P2X_7 _receptor regulates growth of epithelial cells. The specific objective of the present study was to understand to what degree the P2X_7 _system controls development and growth of skin cancer in vivo, and what cellular and molecular mechanisms are involved in the P2X_7 _action.

**Methods:**

Skin neoplasias in mice (papillomas, followed by squamous spindle-cell carcinomas) were induced by local application of DMBA/TPA. Experiments in-vitro utilized cultured epidermal keratinocytes generated from wild-type or from P2X_7_-null mice. Assays involved protein immunostaining and Western blots; mRNA real-time qPCR; and apoptosis (evaluated in situ by TUNEL and quantified in cultured keratinocytes as solubilized DNA or by ELISA). Changes in cytosolic calcium or in ethidium bromide influx (P2X_7 _pore formation) were determined by confocal laser microscopy.

**Results:**

(a) Co-application on the skin of the P2X_7 _specific agonist BzATP inhibited formation of DMBA/TPA-induced skin papillomas and carcinomas. At the completion of study (week 28) the proportion of living animals with cancers in the DMBA/TPA group was 100% compared to 43% in the DMBA/TPA+BzATP group. (b) In the normal skin BzATP affected mainly P2X_7_-receptor – expressing proliferating keratinocytes, where it augmented apoptosis without evoking inflammatory changes. (c) In BzATP-treated mice the degree of apoptosis was lesser in cancer than in normal or papilloma keratinocytes. (d) Levels of P2X_7 _receptor, protein and mRNA were 4–5 fold lower in cancer tissues than in normal mouse tissues. (e) In cultured mouse keratinocytes BzATP induced apoptosis, formation of pores in the plasma membrane, and facilitated prolonged calcium influx. (f) The BzATP-induced apoptosis, pore-formation and augmented calcium influx had similar dose-dependence for BzATP. (g) Pore formation and the augmented calcium influx were depended on the expression of the P2X_7 _receptor, while the BzATP-induced apoptosis depended on calcium influx. (h) The BzATP-induced apoptosis could be blocked by co-treatment with inhibitors of caspase-9 and caspase-3, but not of caspase-8.

**Conclusion:**

(a) P2X_7_-dependent apoptosis is an important mechanism that controls the development and progression of epidermal neoplasia in the mouse. (b) The P2X_7_-dependent apoptosis is mediated by calcium influx via P2X_7 _pores, and involves the caspase-9 (mitochondrial) pathway. (c) The diminished pro-apoptotic effect of BzATP in mouse cancer keratinocytes is possibly the result of low expression of the P2X_7 _receptor. (d) Activation of P2X_7_-dependent apoptosis, e.g. with BzATP could be a novel chemotherapeutic growth-preventive modality for papillomas and epithelial cancers in vivo.

## Background

The current theory of growth of epithelial cells predicts regulation by the concerted effects of mitogenic stimuli and apoptosis [1,2]. Apoptosis is a homeostatic process orchestrated by the host's genome of selective cell deletion without stimulating inflammatory response [3-5]. Dysregulation of apoptotic cell-death has been implicated in states of disease and in the neoplastic transformation [6,7]. Among the pro-apoptotic systems that operate in epithelia [8] the P2X_7 _is an important mechanism because the receptor is expressed by proliferating cells [9], and activation of the receptor induces apoptosis that controls directly growth of the epithelial cells [10].

The P2X_7 _receptor is a membrane-bound, ligand-operated channel [11-13]. The natural ligand of the receptor is ATP [11,12] which is present in the extracellular fluid of epithelial cells at high nanomolar, low micromolar levels [14-18]. In contrast to other types of ATP receptors, activation of the P2X_7 _receptor requires relatively high concentrations of the ligand [12]. However, studies in epithelial cells of the female reproductive tract showed a threshold effect and activation of P2X_7_-mediated apoptosis already by nanomolar concentrations of ATP [8,18], suggesting that ATP levels which are present in the extracellular fluid suffice to activate the receptor.

Binding of the ligand to the P2X_7 _receptor can activate various cell-specific signaling cascades, including the IL-1β [19], TNFα – TRAIL [20], and the p38, JNK/SAPK [21] and NF-κB cascades [22]. However, a unique effect of activation of the P2X_7 _receptor is formation of pores in the plasma membrane [12]. In uterine epithelial cells formation of P2X_7 _receptor pores induces apoptosis by a mechanism that involves uncontrolled influx of Ca^2+ ^via P2X_7_-pores and activation of the mitochondrial – caspase-9 pathway [13,18,23].

Until recently relatively little was known about the biological role of the P2X_7 _in vivo, and particularly in the epidermis. Earlier studies suggested involvement of the P2X_7 _receptor in the inflammatory and immune processes since the receptor is expressed in Langerhans and inflammatory dendritic epidermal cells [24] and in cultured immature dendritic epidermal cells [25]. Overexpression of P2X_7 _was found in lesional skin of psoriasis and atopic dermatitis, where an intense P2X_7 _immunoreactivity was confined to the cell membrane of the basal layer [26]. P2X_7 _may also play a role in chemokine secretion by normal keratinocytes but available data are inconsistent. Inoue et al [27] reported that treatment of cultured normal keratinocytes with the P2X_7 _specific agonist 2',3'-0-(4-benzoylbenzoyl)-adenosine 5'-triphosphate (BzATP) increased IL-6 release, while Pastore et al [26] described that BzATP down-modulated chemokine secretion.

Studies also suggested a role for P2X_7 _in the control of epidermal growth, but most studies were observational. The P2X_7 _receptor is expressed in normal [28], in precancerous epidermal tissues [29], and in skin cancer cells [26,30,31]. P2X_7 _receptors were detected already in 8–11 week-old human fetal epidermis; they colocalized with caspase-3 and with periderm cells positive for transferase-mediated dUTP nick-end-labeling (TUNEL) [32]. Co-localization of the P2X_7 _with apoptosis-related markers was also reported in adult human epidermis [28], and recent studies reported BzATP-induced cell death in normal and cancer keratinocytes, presumably by augmented apoptosis [29,30].

Although existing observational data suggest that the P2X_7 _may regulate growth of epithelial cells, no previous studies investigated experimentally the biological role of the P2X_7 _receptor in vivo. The present study tested the hypothesis that P2X_7 _controls epidermal cell growth in vivo by apoptosis.

## Methods

### Experiments in mice in vivo

The experiments were approved by the Case Western Reserve University (CWRU) Institutional Animal Care and Use Committee (IACUC) protocol [2006-0141], and all experiments were done in accordance with CWRU IACUC policy, following nationally and internationally recognized guidelines. Experiments utilized 6–8 weeks old wild-type FVB female mice (Charles River, Wilmington, MA). The animal's dorsal skin (~3 × 5 cm) was shaved using Oster animal clipper (Mountain Home, AR) followed by weekly application of Nair hair remover lotion http://www.naircare.com. Skin neoplasia in the mice were induced by the "Two-Step" method, which involved tumor initiation by local treatment with 7,12-dimethyl-benz(a)anthracene (DMBA), followed by tumor promotion with local treatment of 12-O-tetradecanoylphorbol-13-acetate (TPA) [e.g. [33]].

Treatments included the application of one or more of the following drugs, directly onto the shaved dorsal skin: DMBA (50 μg/200 μl acetone [950 μM, 190 nmol, 3.3 μg/cm^2^]); TPA (3 μg/200 μl acetone [20 μM, 4 nmol, 0.2 μg/cm^2^]); BzATP (14.3 μg/200 μl of 2.3/1 vol/vol solution of propylene-glycol [PG]/ethanol [EtOH] [100 μM, 20 nmol, 1.0 μg/cm^2^]); or the BzATP vehicle only (200 μl of PG/EtOH). DMBA was applied once, while TPA and BzATP were applied twice a week. The concentration/dose of BzATP and frequency of treatments were chosen based on data in cultured cells [8,23].

The First experiment (Experiment-1) included three groups of animals: Control (no treatment, n = 13); DMBA plus TPA (n = 15); and DMBA/TPA plus BzATP (n = 12). Treatments with BzATP began two weeks prior to the DMBA/TPA treatments. Endpoints were determined at 0–12 (Papilloma Phase) and 14–28 weeks (Cancer Phase) after DMBA. Endpoints were the incidence and prevalence of lesions, as well as the mean lesion size in each living animal. Biopsy of skin papilloma was done in one living animal using mini rotary ElliptiPunch blade (HUOT, Menomonee Falls, WI). Criteria for euthanasia (by cervical dislocation) prior to week 28 were according to Montgomery guidelines [34], including animals with excessive tumor burden, or with ulcerated lesions regardless of size that were bleeding, necrosed, or infected. The experiment was terminated at week 28, when all remaining living animals were euthanized. After death, representative samples were obtained from all skin lesions as follows: From the DMBA/TPA group 4 papillomas and 26 cancer samples; and from the DMBA/TPA-BzATP group 2 papillomas and 12 cancer samples. All animals underwent postmortem exam for the presence of metastases and non-epidermal tumors (none were found).

Lesions developed at 0–12 weeks were defined papillomas based on the actual histological diagnosis in one case, the typical morphological appearance, and the extensive experience gained by others using this model and methodology [33,35]. Lesions developed at 14–28 weeks were defined either as cancers (as diagnosed in each case at the time of death), or as non-cancerous lesions, including existing or involuting papillomas (diagnosed at the time of death) or lesions that disappeared prior to death.

Experiments 2 and 3 studied the effects of BzATP in normal mice. Experiment-2 included one group of five animals. The shaved dorsal skin was divided into equal anterior and posterior areas. Each animal was treated with BzATP, applied locally twice a week for 4 weeks on the anterior skin area; vehicle only was applied in parallel twice a week for 4 weeks on the posterior skin area. At the end of the experiment animals were euthanized and strips were obtained from each animal from the anterior and posterior treated dorsal skin areas.

Experiment-3 included two groups of 5 normal animals, Control (vehicle only) and BzATP. Animals were treated for 16 weeks, and after euthanasia strips were obtained from each animal from the treated dorsal skin areas. In addition, blood samples were obtained from each animal from the retro-bulbar venous complex for plasma alanine aminotransferase (ALT, glutamic pyruvic transaminase [GPT]), and aspartate aminotransferase (AST, glutamic oxalacetic transaminase [GOT]) assays.

Skin tissues from animals of Experiments 1–3 were assayed for histology (H&E), P2X_7_-receptor immunoreactivity, and in-situ TUNEL.

### Experiments using cultured mouse keratinocytes

The experiments utilized primary cultures of epidermal keratinocytes generated from 6–8 weeks old wild-type C57Bl mice (Charles River, Wilmington, MA); from P2X_7_-/-Pfizer mice [36], constructed by deletion of amino acids 506–532 of the C-terminus which is critical for P2X7-mediated apoptosis [12,23]; or from P2X_7_-/-GSK mice, which have a *lacZ *gene inserted at the beginning of exon 1, resulting knockout of the receptor, [37]. The P2X_7_-/-Pfizer and P2X_7_-/-GSK mice were generated on the C57Bl background.

Primary cultures of mouse keratinocytes were generated by the collagenase-EDTA method as described [38], with minor modifications. Ventral superficial skin areas involving the full epidermis and part of the dermis were scraped and the cell suspension was washed by PBS and filtered through a 40-μm cell strainer. Following repeated washes and spinning cells were plated at a density of 1 × 10^5 ^cells per cm^2 ^on type-I collagen-coated filters [23] and used for experiments after 24–48 hours.

P2X_7_-specific antisense oligonucleotides (ASO) and random control oligonucleotides (RCO) were designed from the published sequence of the mouse P2X_7 _gene [39] (AJ009823) using a previously described method [40]. The sequences of the 20-mer ASO that would hybridize to the coding region of exon 13 (nt 1467–1486), and the RCO were as follows: ASO – GGC GTA CCG CAG CAA CGT AG; RCO – TAA GTA CTG CAG CTA CGT AC (designed such that no cross-hybridization against the P2X_7 _gene would occur). To assess the effects of the ASO and RCO on P2X_7 _mRNA expression, cultured cells were treated for 14 hours with or without 100 μM ASO or RCO.

### Protein and mRNA methods

The receptor P2X_7 _protein in tissues cross sections was detected by immunostaining as described [9,38]. The specificity of staining was previously shown in terms of incubations in the absence or presence of the P2X_7 _antigen peptide, where co-incubation with the P2X_7 _antigen peptide blocked P2X_7 _immunoreactivity [9]. Immunofluorescence was captured in a fluorescence microscope Nikon Eclipse 80i (Nikon, Melville, NY), and image analysis of the immunofluorescence data was described [9,38]. Briefly, fields of interest were captured and saved in Adobe Photoshop. Pictures were scanned using UN-SCAN-IT software (Silk Scientific, Orem, UT) by choosing 5 representative fields for each picture. Fields of interest were chosen in reference to the epithelial component of the tissue, as determined by switching to phase microscopy of the same slide. Light intensity in each field was digitized, and average pixel density for P2X_7 _per field was determined using the program software.

Western blotting of cell lysates using the anti-P2X_7 _antibody, and densitometry of the P2X_7_-specific 75 KDa band (relative to glyceraldehyde-3-phosphate dehydrogenase, GAPDH) were done as was previously described [23].

Total RNA was extracted by RNeasy mini kit (Qiagen, Valencia, CA), and two-step real-time qPCR was carried out as described [23]. The following mouse-specific primers were used: P2X_7 _receptor, forward 5'-TTC CAG GAA GCA GGA GAG AA-3', reverse 5'-ATA CTT CAA CGT CGG CTT GG-3' (annealing at 58°C, 2 min). GAPDH, forward 5'-TGT TGC CAT CAA TGA CCC C-3', reverse 5'-ATG AGT CCT TCC ACG ATA CC-3' (annealing at 61°C, 1 min). Relative quantification (RQ) was calculated using Applied Biosystems SDS software (Foster City, CA) based on the equation RQ= 2^-ΔΔCt ^where C_t _is the threshold cycle to detect fluorescence.

### Apoptosis assays

TUNEL assays in tissues cross sections were performed using DeadEnd™ Fluorometric TUNEL System (Promega, Madison, WI) according to the manufacturer's protocol. For TUNEL-P2X_7 _co-staining, tissue cross sections were first assayed for TUNEL, followed by P2X_7 _immunostaining. The modified combined method resulted in only negligible cross fluorescence interference.

Apoptosis of cultured mouse keratinocytes was quantified in terms of percent solubilized DNA [8], or by using the commercial cell-death detection ELISA kit (Roche Applied Science, Nutley, NJ) [23].

### ALT and AST assays

ALT and AST assays used Liquid Reagent kits (Pointe Scientific, Canton MI), and were performed according to the supplier instructions.

### Cytosolic Calcium (Ca^2+^_i_) and Ethidium-Bromide assays

Changes in Ca^2+^_i _in cultured cells were determined in terms of changes in intracellular Fluo-4 fluorescence using dynamic confocal laser scanning microscopy as was previously described [23]. Cultured mouse keratinocytes were loaded with 5 μM Fluo-4/AM, and imaged with a Zeiss LSM 510 inverted real-time confocal microscope [23]. Images were collected at 488 nm/505 nm (exc/emi) at intervals of 10 to 15 seconds after treatment with 100 μM BzATP, added to the perfusate. For ethidium bromide influx experiments, glass-bottomed dishes cultured with mouse keratinocytes were placed in the microscope. Images (collected at 488 nm/505 nm [exc/emi]) were taken before, and at intervals of 30 seconds after adding 5 μM ethidium bromide to the perfusate as described [23]. Average fluorescence intensity was quantified from collated images using MetaVue softeware (Fryer Company Inc., Huntley, IL) by subtracting the basal intensity value [23].

### DNA synthesis assay

Changes in DNA synthesis were determined in terms of [^3^H]thymidine incorporation as described [10]. The radioactivity (dpm/mg Protein, determined by Bio-Rad Protein Assay solution [Hercules, CA]) of triplicated samples was determined by beta scintillation counting (Beckman LS1801 scintillation counter).

### Data Analysis

The proportion of living animals with papillomas or cancerous lesions in Experiment 1 was calculated at each week throughout the study period and compared between groups by chi-square or Fisher's exact test. Time-to-event data were used to assess differences in the formation of cancerous lesions and the Kaplan-Meier method (with log-rank test) was used to compare differences between groups. Mean number of lesions per animal was calculated at each week and compared between groups by independent samples t-test. In addition, repeated-measure ANOVA was used to examine the effect of time and number of lesions between groups. Mean lesion size was compared similarly for weeks 0–12. For weeks 14–28, non-cancerous and cancerous lesions were categorized as ≤ 10 mm^3 ^versus > 10 mm^3 ^and the proportion of lesions > 10 mm^3 ^in living animals was compared between groups by χ^2^-square or Fisher's exact test. Cancerous lesions were also categorized as ≤ 200 mm^3 ^versus > 200 mm^3^. Cancer development (for weeks 14–28) and Survival rates (for weeks 0–28) were evaluated using the Kaplan-Meier method and compared between groups by log-rank test. For cultured cells data, significance of differences between groups was estimated by t-test, or by one-way or two-way ANOVA with Tukey-Kramer Multiple Comparisons post test analysis.

### Supplies

All chemicals, unless specified otherwise, were obtained from Sigma Chemical (St. Louis, MO). Primary antibodies for the immunostaining and Western blots were as follows: Rabbit polyclonal anti-P2X_7 _receptor antibody, which recognizes the functional full length P2X_7 _receptor [23] was from Alomone Laboratories (Jerusalem, Israel); rabbit anti-GAPDH antibody was from BD Transduction Laboratories (Lexington, KY). The secondary antibody was goat anti-rabbit Alexa Fluro antibody (Invitrogen, http://www.invitrogen.com) [23]. Leu-Glu-His-Asp-O-methyl-fluoromethylketone (LEHD-FMK), Ile-Glu-Thr-Asp-O-methyl-fluoromethyketone (IETD-FMK), Benzyloxy-valine-alanine-aspartate-O-methyl-fluoromethylketone (zVAD-FMK), and Asp-Glu-Val-Asp-O-methyl-fluoromethylketone (DEVD-FMK) were from Calbiochem (La Jolla, CA), and were used at a concentration of 50 μM.

## Results

### BzATP modulates DMBA/TPA skin effects (Experiment-1)

**Figure 1:**
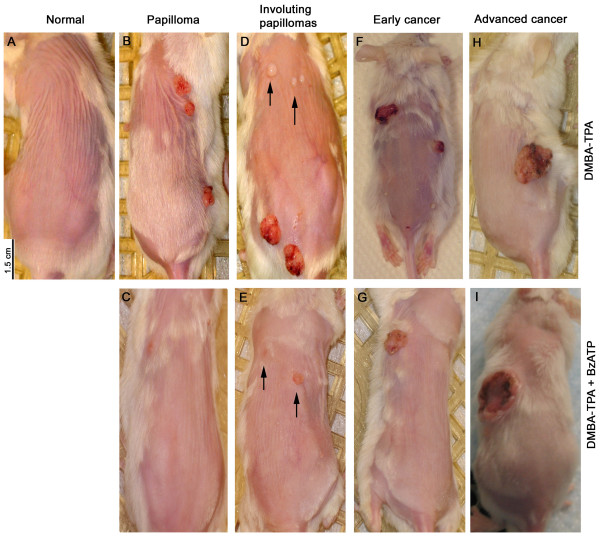
**Representative pictures of DMBA/TPA – induced skin lesions in mice in-vivo, and the effects of co-treatment with BzATP**. Arrows in **D **and **E **point to involuting papillomas.

**Figure 2:**
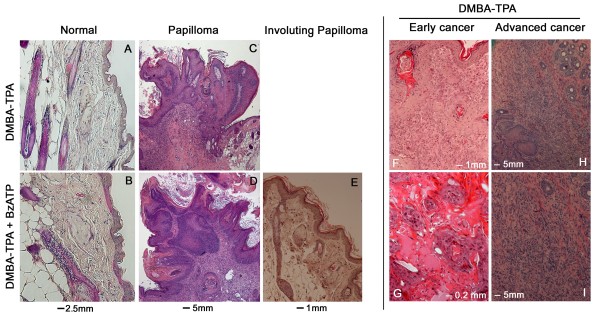
**Representative cross-sections, evaluated histologically by H&E, of DMBA/TPA – induced skin lesions in mice in-vivo, and the effects of co-treatment with BzATP**.

**Figure 3:**
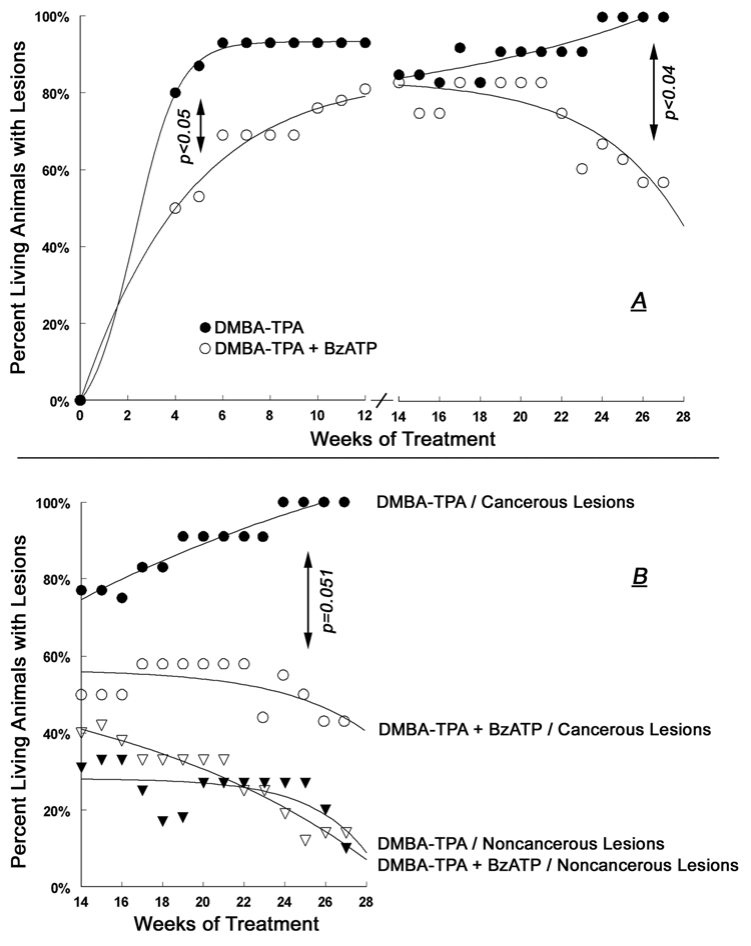
**Summary of the effects of local treatments with DMBA/TPA (black symbols) or DMBA/TPA+BzATP (white symbols) on the proportion of living mice with skin lesions (Experiment-1)**. In **A**, skin lesions at 0–12 weeks of treatment were papillomas. In **A **and **B**, skin lesions at 14–28 weeks of treatment were grouped either as cancerous lesions (squamous spindle-cell carcinomas, circles), or as non-cancerous lesions (existing or involuting papillomas, triangles). Values in **A **and **B **are means; standard deviation (SD) ranged 3–11%.

**Figure 4:**
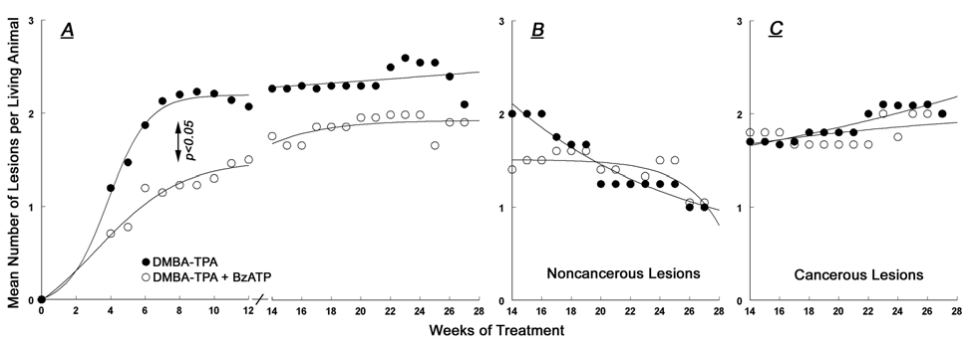
**Summary of the effects of local treatments with DMBA/TPA (black symbols) or DMBA/TPA+BzATP (white symbols) on the mean number of skin lesions per living animal (Experiment-1)**. Definitions of lesions were as in Fig. 4. Values in **A-C **are means; SD ranged 5–9%.

**Figure 5:**
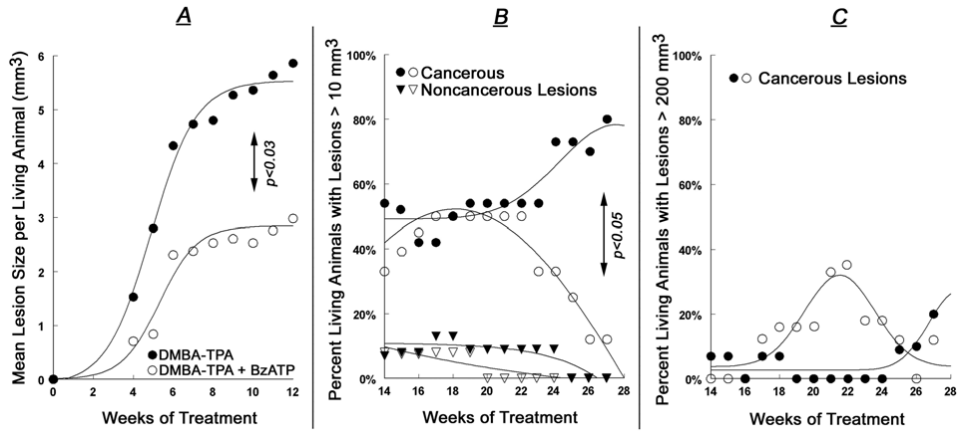
**Summary of the effects of local treatments with DMBA/TPA (black symbols) or DMBA/TPA+BzATP (white symbols) on the mean lesion size at 0–12 weeks of treatment (A); and on the proportion of living mice with total lesions volume per animal of > 10 mm^3 ^(B) or > 200 mm^3 ^(C) (Experiment-1)**. Definitions of lesions were as in Fig. 3. Values in **A-C **are means; SD ranged 2–18%.

**Figure 6:**
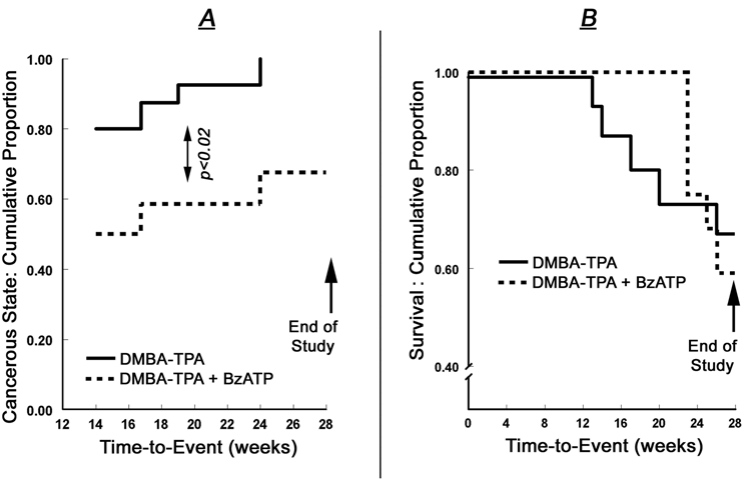
**Summary of the effects of local treatments with DMBA/TPA (black symbols) or DMBA/TPA+BzATP (white symbols) on the time-to-event of cancer state (A) and on the animals' survival rates (B) (Experiment-1)**. Definitions of lesions were as in Fig. 3.

Local administration of DMBA/TPA induced formation of skin lesions, papillomas at weeks 5–12, and squamous spindle-cell carcinomas afterwards (Figs. 1, 2). About one thirds of the papillomas involuted after week 14 and the remaining persisted either as non-cancerous papillomas, or have transformed to cancerous lesions (Figs. 1, 2, 3, 4, 5, 6). All cancerous lesions arose from pre-existing papillomas. None of the animals in the control group had developed skin lesions (Fig. 1A).

Co-treatment with BzATP, applied locally on skin areas exposed to DMBA/TPA altered the incidence and pattern of skin lesions (Figs. 1, 2, 3, 4, 5, 6). To evaluate the effects of BzATP, changes in skin lesions in the DMBA/TPA and DMBA/TPA+BzATP groups were compared relative to the length of treatment. Since formation of papillomas and cancerous lesions was time-related, with a marked cut-off at weeks 13–14 (Fig. 3A), data were analyzed separately for weeks 0–12 and 14–28.

#### Proportion of animals with lesions

At weeks 0–12 the proportion of living animals with papillomas tended to be lower in the DMBA/TPA+BzATP group than in the DMBA/TPA group, and analysis of the proportion having a papilloma separately gave a significant (p < 0.05) difference at week 5 of treatment (Fig. 3A; 48 ± 12% versus 80 ± 10%, respectively). The proportion of living animals with any skin lesion at weeks 14–21 was similar in the two groups, being initially influenced by the death of two animals with cancers in the DMBA/TPA group at weeks 13–14 (Fig. 3A, filled circles). However, the proportion of living animals with any skin lesion differed significantly among the groups in weeks 22–28 (Fig. 3A). During that period of time the proportion of living animals with non-cancerous lesions (existing and involuting papillomas) decreased in both groups (Fig. 3B). In contrast, the proportion of living animals with cancerous lesions in the DMBA/TPA group increased steadily while in the DMBA/TPA+BzATP group it decreased over time (Fig. 3B). For example, in week 28 the proportions of living animals with cancerous lesions in the DMBA/TPA and the DMBA/TPA+BzATP groups were 100% and 43 ± 9%, respectively (Fig. 3B).

#### Mean number of lesions

In both groups the mean number of papillomas per living animal increased at weeks 0–12, but the increase in the DMBA/TPA+BzATP group tended to be smaller than in the DMBA/TPA group (Fig. 4A). Independent samples t-test revealed a significant difference at week 10 (2.3 ± 0.5 and 1.2 ± 0.4 papillomas per animal [mean ± SD], respectively, p < 0.04). Also, repeated measures ANOVA yielded a significant time effect (p < 0.01) for the DMBA/TPA and DMBA/TPA+BzATP curves at weeks 0–12 (Fig. 4A). At weeks 14–28 the mean number of total lesions per living animal was not significantly different between the two groups (Fig. 4A). In both groups the mean number of non-cancerous lesions decreased over the 14–28 weeks period (Fig. 4B), while the mean number of cancerous lesions remained the same (Fig. 4C).

#### Mean lesion size

Animals in both groups were compared relative to the total size of lesions (in mm^3^) per animal. In both groups the mean total papillomas size per living animal increased at weeks 0–12, but the increase in the DMBA/TPA+BzATP group was smaller than in the DMBA/TPA group (Figs. 1B, C, 5A). Independent samples t-test revealed significant differences at all weeks for mean total papillomas size (p < 0.01–0.03, Fig. 5A). For example, in week 12 mean total papillomas size (in mm^3^) per animal was 5.8 ± 1.1 versus 3.4 ± 1.0 (mean ± SD), respectively (p < 0.01, Fig. 5A). Likewise, repeated measures ANOVA yielded a significant time effect (p < 0.01); a significant group effect (p < 0.02); and a non-significant time*group interaction effect (p > 0.1), for the DMBA/TPA and DMBA/TPA+BzATP curves (Fig. 5A). The latter analysis indicates non-interacting trends for the two curves.

At weeks 14–28 the variability of the lesions sizes among the two groups was large, due to the excessive growth of some lesions unproportionally to others (e.g. Figs. 1D–I). This precluded us from comparing means of lesion size among the two groups. However, since most non-cancerous lesions in both groups tended to be smaller than 10 mm^3 ^and the proportion of animals with non-cancerous lesions of > 10 mm^3 ^was low (< 10%) in both groups (Fig. 5B, triangles), data of the proportion of living animals with cancerous lesions > 10 mm^3 ^were compared among the two groups. Figure 5B shows a significantly smaller proportion of living animals in the DMBA/TPA+BzATP group with cancerous lesions > 10 mm^3 ^after week 23 than in the DMBA/TPA group. For example, in week 28 the proportion of living animals with cancerous lesions > 10 mm^3 ^were 81% ± 8% compared to 16 ± 4% in the DMBA/TPA and the DMBA/TPA+BzATP groups, respectively (Fig. 5B).

Interestingly, five mice in the DMBA/TPA+BzATP group survived despite having developed relatively large cancerous lesions (e.g. Fig. 1I), while maintaining normal weight and exhibiting normal behavior. In contrast, most mice in the DMBA/TPA group with already smaller cancerous lesions (e.g. Fig. 1H) had to be euthanized per protocol due to poor general condition and excessive tumor burden. Analysis of the proportion of living animals with cancerous lesions > 200 mm^3 ^showed a tendency for higher proportion of animals in the DMBA/TPA+BzATP group than in the DMBA/TPA group (Fig. 5C), but the differences did not reach statistical significance.

#### Summary of the trends of cancer development and survival rates

Using time-to-event data analysis it was found that development of cancerous lesions was significantly slower and lower in the DMBA/TPA+BzATP group than in the DMBA/TPA group (Fig. 6A).

Survival curves for the DMBA/TPA and DMBA/TPA+BzATP groups were generated based on event (death from cancer) and time-to-event (in weeks) for each group. Log-rank test was used to compare the survival curves based on group. The overall survival rates among the two groups did not differ statistically, although there was a tendency of earlier deaths in the DMBA/TPA group compared to the DMBA/TPA+BzATP group (Fig. 6B).

#### Animals' weights

There were no significant differences in animals' weights among the control or the DMBA/TPA and DMBA/TPA+BzATP treatment groups over the course of 28 weeks (not shown).

#### Morphological and histological skin changes

There were no significant differences in the morphological (Figs. 1A–I) and histological characteristics (Figs. 2A, B) of the unaffected normal skin in the DMBA/TPA and the DMBA/TPA+BzATP groups. Similarly there were no significant differences in the morphological (Figs. 1B–E) and histological characteristics of papillomas in the DMBA/TPA and DMBA/TPA+BzATP groups (Figs. 2C, D). In both groups after week 14 some papillomas remained intact while other started to involute (Figs. 1D, E, 2E). However, in both groups most papillomas (about two third) underwent cancerous transformation to squamous cell carcinomas with spindle-cell changes (Figs. 2F–I). There were no significant changes in the morphological (Figs. 1F–I) and histological characteristics (not shown) of cancers in the two groups.

### P2X_7 _receptor expression is lower in mouse skin cancer tissues

**Figure 7:**
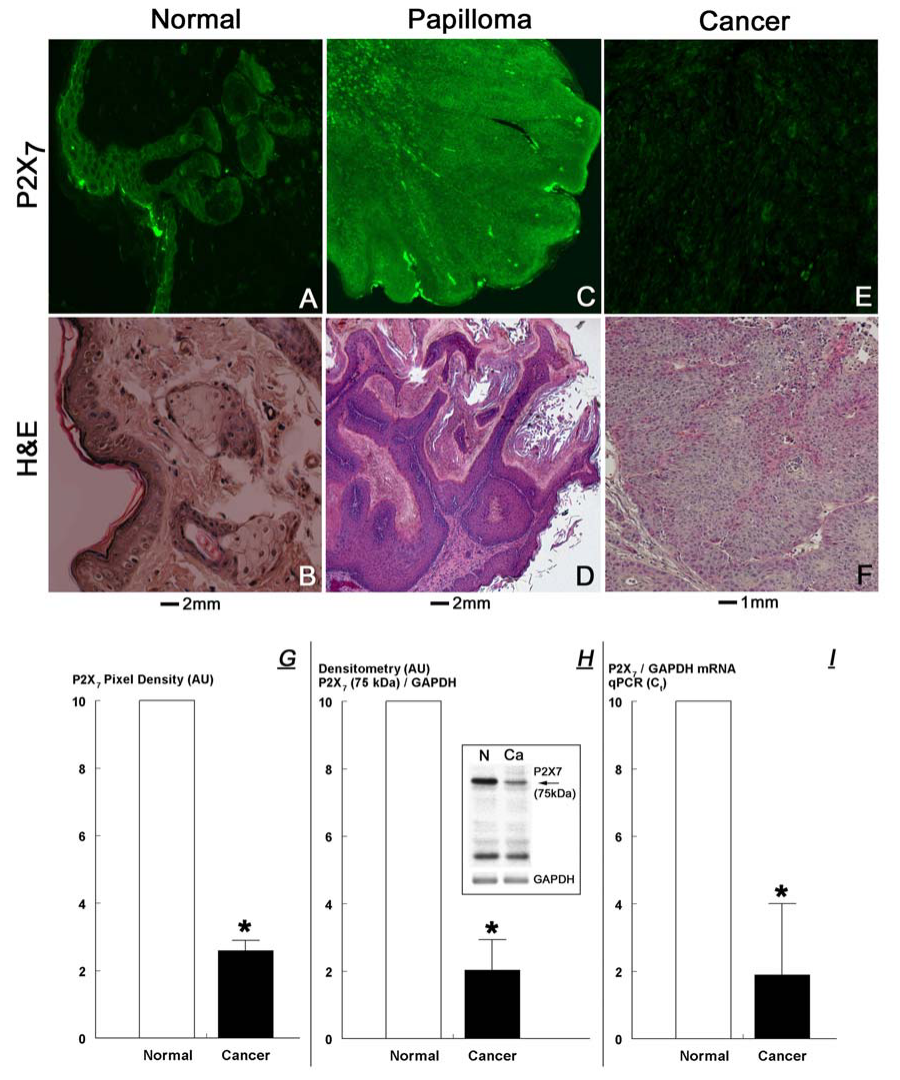
**P2X_7 _immunoreactivity in mouse normal skin (A), papilloma (C), and skin cancer tissues (E) (Experiment-1); B, D, F are parallel cross sections, respectively, stained by H&E**. **G**. Analysis of P2X_7 _immunoreactivity compared among paired histologically normal and cancerous tissues. Bars are means (± SD) of levels in tissues of five mice. **H**. P2X_7 _protein assays in mouse normal and cancer skin tissues. Lysates fractionated by gel electrophoresis were immunoblotted with the anti P2X_7 _antibody and membranes were reprobed with the anti GAPDH antibody. Insert in **H **shows Western immunoblot of lysates of histologically normal and cancerous tissues obtained from the same animal. Similar results were obtained in tissues of two additional mice. Bars show means (± SD) of densitometry results of the P2X_7_-specific 75 KDa bands in tissues of three mice. **I**. P2X_7 _mRNA levels (relative to GAPDH mRNA) (means ± SD) in histologically normal and cancerous tissues obtained from three animals. In **G-I **data in the normal tissues were normalized in each case to an arbitrary value of 10. * – p < 0.01. AU – arbitrary units.

Cellular effects of BzATP are mediated mainly by the P2X_7 _receptor [11,12]. The present data showed that mouse normal, papilloma, and cancer skin cells express the P2X_7 _receptor, but levels of the receptor in cancer tissues were significantly lower than in normal skin or papilloma tissues (Fig. 7). Immunostaining with the anti P2X_7 _receptor antibody of tissue cross sections containing normal skin revealed intense immunoreactivity that localized predominantly in the epidermis within proliferating keratinocytes and epidermal hair shafts (Figs. 7A, B). In papillomas, P2X_7 _immunoreactivity was intense (Fig. 7C), similar to normal tissues (Fig. 7A), and it localized predominantly within proliferating keratinocytes at the base of the developing papillomas (Figs. 7C, D). In contrast, P2X_7 _immunoreactivity in cancer tissues was significantly lesser than in normal epidermal or papilloma tissues (Figs. 7E, F), and data analysis of the P2X_7 _immunostaining revealed a four fold lesser P2X_7 _immunoreactivity in cancer than in normal tissues (Fig. 7G).

The immunostaining data were confirmed by Western blot experiments. Assays of the P2X_7_-specific 75 KDa band revealed a five fold lower density in cancer tissues than in normal tissues (Fig. 7H). Further confirmation was obtained by P2X_7 _mRNA experiments where qPCR assays revealed a five fold lower P2X_7 _mRNA/GAPDH mRNA levels in normal tissues than in cancer tissues (Fig. 7I). Collectively, the data in Fig. 7 indicate that P2X_7 _receptor expression levels in mouse skin cancer tissues are four-five fold lower than in mouse normal skin tissues.

### BzATP augments apoptosis in the normal skin (Experiments 2 and 3)

To better understand the cellular effects of BzATP in vivo, experiments investigated the effects of BzATP in the normal mouse on skin morphology and histology; on the immunoreactivities with the P2X_7 _antibody; and on apoptosis.

**Figure 8:**
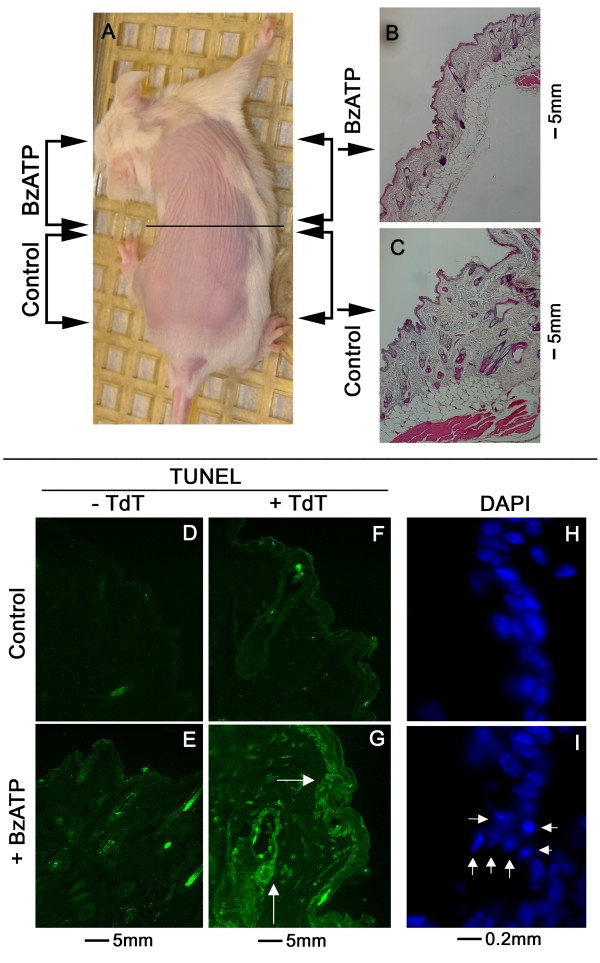
**Effects in mice in-vivo of local treatment with BzATP on skin apoptosis (Experiment-2)**. **A**. Mice (n = 5) were treated with BzATP, applied locally twice a week for 4 weeks on the shaved anterior skin area, and with the vehicle (Control) applied in parallel on the shaved posterior skin area. The horizontal line shows schematically separation of the anterior and posterior dorsal skin regions. At the end of the experiment animals were euthanized and skin cross sections were generated from each animal from the anterior (**B**, BzATP) and posterior (**C**, Control) dorsal skin areas for H&E (**B, C**), TUNEL (**D-G**), and DAPI (**H, I**) staining. **D-G**: TUNEL staining of BzATP-treated skin tissues (**E, G**) and of Control skin tissues (**D, F**), processed in the absence (**D, E**) or the presence (**F, G**) of the TUNEL active reagent TdT (Promega). Arrows in **G **show enhanced TUNEL staining in epidermal cells of the basal/parabasal layers (horizontal arrow) and of epidermal hair shaft cells (vertical arrow). **H, I**: DAPI (nuclear) staining. Arrows in **I **point to nuclei of epidermal cells in basal/parabasal regions of the epidermis at advanced stages of condensation, fragmentation and pyknosis. Data in A-I are representative of similar results in five animals.

In animals of Experiment-2, BzATP was applied twice weekly for 4 weeks on the anterior region of the shaved dorsal skin and each animal served as its own control by having vehicle-containing solution applied twice weekly for 4 weeks on the posterior region of the shaved dorsal skin (Fig. 8A). Treatments with BzATP or the vehicle solution had no visible morphological effect on the skin (Fig. 8A), and histological evaluation showed no differences in cross sections obtained from the BzATP-treated or control skin areas (Figs. 8B, C).

Experiment-2 also studied the effects of local treatment with BzATP on skin apoptosis in vivo by TUNEL staining. The negative control experiment showed minimal auto-fluorescence in cross sections of the mouse skin (Figs. 8D, E). In skin cross sections of non-treated mice, only faint TUNEL staining decorated the epidermis (Fig. 8F). In contrast, in skin cross sections of BzATP-treated mice numerous epidermal basal/parabasal cells and epidermal hair shaft cells stained TUNEL positive (Fig. 8G). Additionally, DAPI stains of cross sections of BzATP-treated skin revealed greater proportion of nuclei at advanced stages of condensation, fragmentation and pyknosis (Fig. 8I) compared to controls (Fig. 8H).

**Figure 9:**
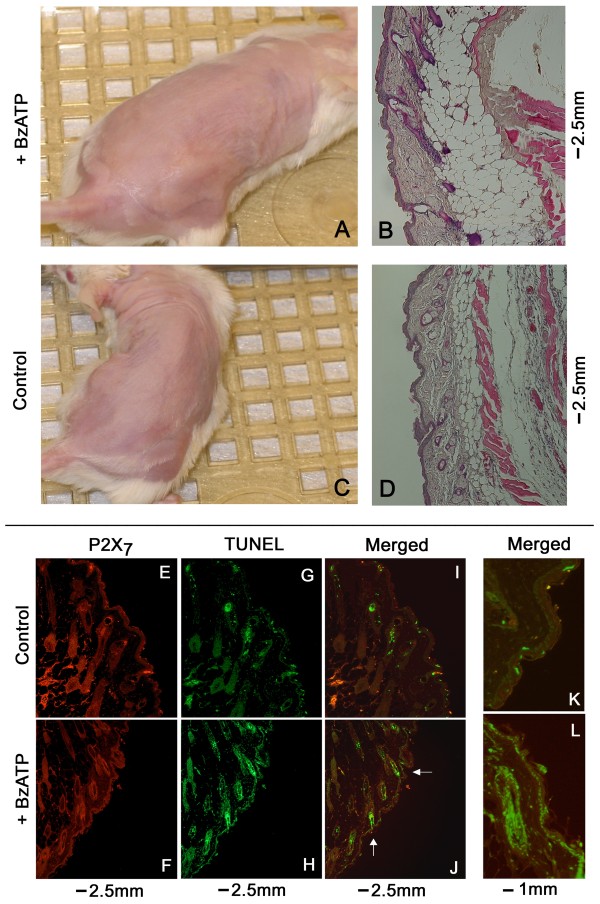
**Effects in mice in-vivo of local treatment with BzATP on skin apoptosis (Experiment-3)**. Animals (n = 5) were treated for 16 weeks either with BzATP, applied locally twice a week on the shaved dorsal skin (**A, B**) or with the vehicle (Control, n = 5, **C, D**). At the end of the experiment animals were euthanized and strips were obtained from each animal dorsal skin areas for H&E (**B, D**) and P2X_7_/TUNEL co-staining (**E-L**). Arrows in **J **show increased TUNEL staining co-localizing with P2X_7 _immunoreactivity in epidermal cells of the basal/parabasal layers (horizontal arrow) and of epidermal hair shaft cells (vertical arrow). Assays were repeated 3–5 times with similar trends.

In animals of Experiment-3, BzATP was applied twice weekly for 16 weeks on the entire shaved dorsal skin. The control group included mice that were treated only with the vehicle. Similar to Experiment-2, treatment with BzATP had no significant effect on skin morphology (Figs. 9A, C) and histology (Figs. 9B, D), compared to treatment with the vehicle only. Also, treatment with BzATP had no significant effect on P2X_7 _immunoreactivity (Figs. 9E, F). However, treatment with BzATP increased the number of TUNEL stained epidermal basal/parabasal and hair shaft cells (Figs. 9G, H), similar to the result in Experiment-2. P2X_7_-TUNEL co-staining showed that the increased TUNEL staining co-localized with P2X_7 _immunoreactivity (Figs. 9I, J [low magnification], Figs. 3K, L [higher magnification]).

Collectively, the data in Fig. 9 indicate that treatment with BzATP, applied locally twice a week on the shaved dorsal skin of normal mice, up-regulated apoptosis of proliferating epidermal and hair shaft keratinocytes. However, in the normal mouse skin the BzATP treatment and the augmented apoptosis did not affect morphology or histology of the skin.

Treatments with BzATP for 4 weeks (Experiment-2) or for 16 weeks (Experiment-3) had no significant effects on the behavior of the animals, on their feeding habits, or on their body weight (not shown). In addition, in animals of Experiment-3 mean ALT and AST plasma levels were similar among the BzATP and control groups, and were in the normal range for the mouse (not shown).

### Effects of BzATP on P2X_7 _expression and TUNEL in papilloma and cancer tissues

**Figure 10:**
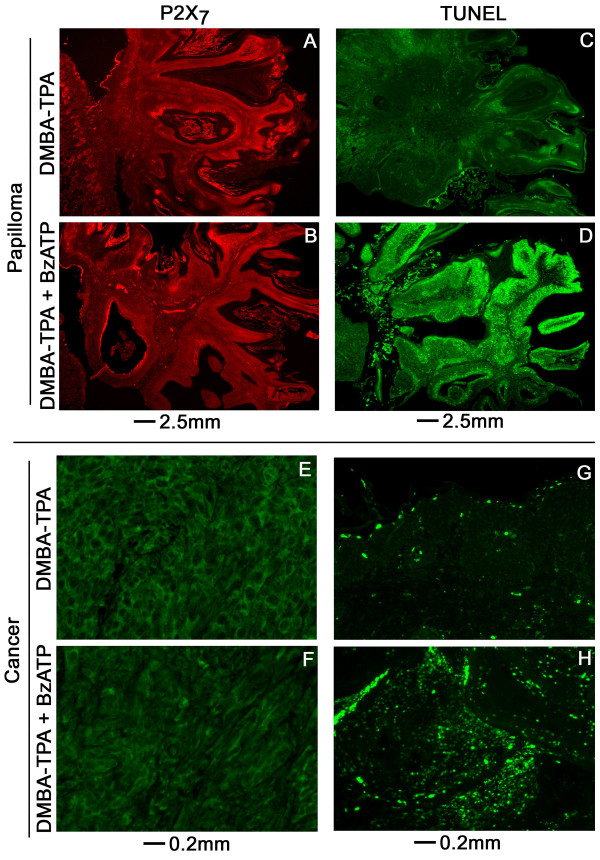
**Effects of treatments with BzATP on P2X_7 _expression and apoptosis in DMBA/TPA – induced skin papillomas (A-D) and cancers (E-H) (Experiment-1)**. Assays were repeated 4 times with similar trends. **C, D, G, H **are parallel cross sections to **A, B, E, F**, respectively.

P2X_7 _immunoreactivities in cross sections of papillomas did not differ in intensity among specimens obtained from DMBA/TPA- or DMBA/TPA+BzATP – treated mice (Figs. 10A, B). The intensity of P2X_7 _immunoreactivity in cross sections of skin cancers was significantly weaker than in normal (Fig. 7A) and in papilloma tissues (Figs. 10A, B), but it did not differ among the DMBA/TPA and DMBA/TPA+BzATP groups (Figs. 10E, F).

TUNEL staining was weak in cross sections of papillomas (Fig. 10C) and cancer tissues (Fig. 10G) from the DMBA/TPA group, similar to findings in cross sections of normal skin (Figs. 8F, 9G). In contrast, TUNEL staining was more intense in cross sections of papillomas (Fig. 10D) and cancer tissues (Fig. 10H) from the DMBA/TPA+BzATP group. In papillomas obtained from mice of the DMBA/TPA+BzATP group, enhanced TUNEL staining decorated basal/parabasal layers of keratinocytes outgrowing at the base of the developing papilloma (Fig. 10D).

### Mechanism of BzATP-augmented apoptosis in mouse keratinocytes

#### Dependence of BzATP-augmented apoptosis on the expression of the P2X_7 _receptor

To better understand the mechanism of BzATP pro-apoptotic skin effects, experiments utilized cultured primary mouse keratinocytes that were obtained from wild-type mice, and from the P2X_7_-receptor – deficient P2X_7_-/-Pf and P2X_7_-/-GSK mice.

**Figure 11:**
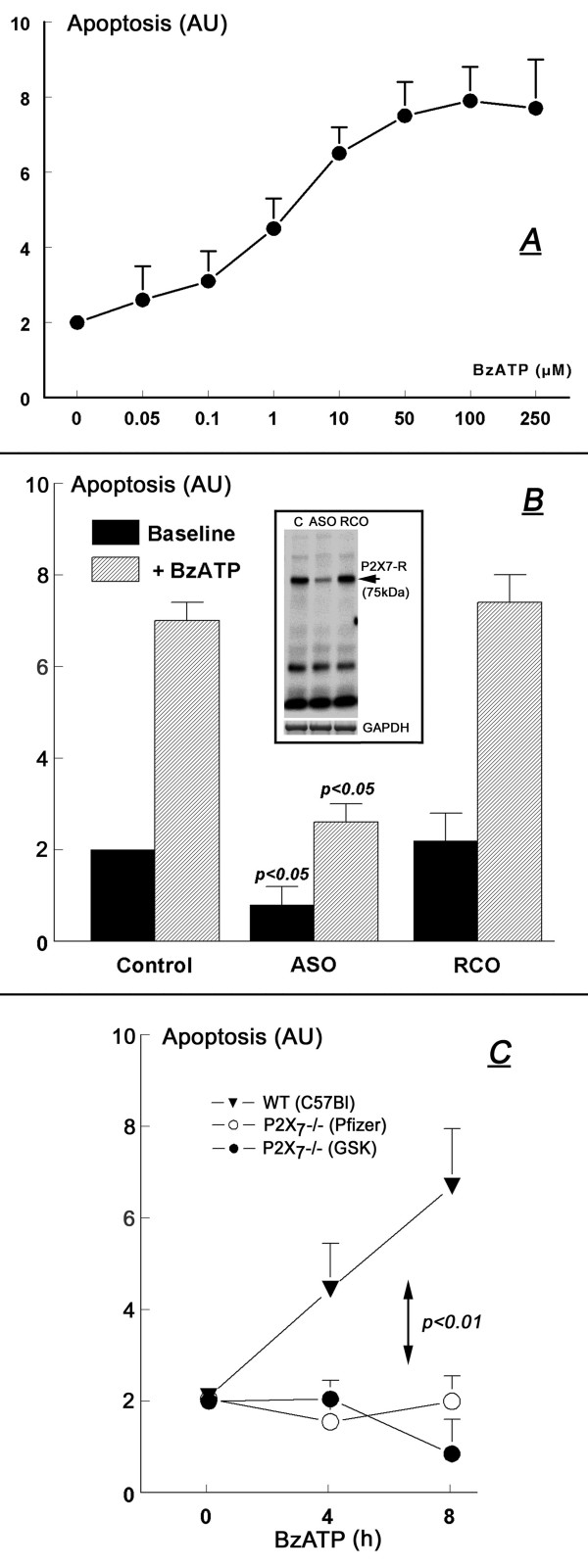
**Effects of BzATP on apoptosis in cultured mouse keratinocytes**. In all experiments levels of apoptosis were normalized to an arbitrary value of 2 in control cells. AU – arbitrary units. **A**. BzATP dose-response effect in cultured mouse normal (wild-type, C57Bl) keratinocytes (means ± SD, n = 3). Cells were treated with one of the indicated concentrations of BzATP for 8 hours. **B**. Cultured mouse normal keratinocytes (wild-type, C57Bl) were pre-treated with 100 μM anti-sense P2X_7 _oligonucleotides (ASO) or random-control P2X_7 _oligonucleotides (RCO) for 14 hours followed by 8 hours treatment with 100 μM BzATP. Control – cells treated with the vehicle of the ASO. Values are means (± SD) of 3 experiments for each condition. Insert in **B **is Western immunoblot with anti-P2X_7 _antibody of lysates of cells treated with ASO or RCO (n = 2). **C**. BzATP time-response effect in cultured mouse normal keratinocytes (wild-type, C57Bl; filled triangles), or in keratinocytes obtained from P2X_7_-deficient (P2X_7_-/-Pfizer [empty circles] or P2X_7_-/-GSK [filled circles]) mice (both in the C57Bl background). Values are means (± SD) of 3 experiments for each condition. Changes in apoptosis in **A **and **B **were determined in terms of solubilized DNA; changes in apoptosis in **C **were determined using cell-death detection ELISA.

In wild-type mouse keratinocytes BzATP augmented apoptosis in a dose-related manner; effects began at BzATP levels as low as 50 nM, reaching maximal effect at 100–250 μM with an estimated BzATP EC_50 _of about 10 μM (Fig. 11A).

Pre-treatment with P2X_7_-receptor anti-sense oligonucleotide decreased expression of the P2X_7_-receptor (Fig. 11B, insert); it also inhibited baseline apoptosis (which most likely is induced paracrinologically by ATP secreted by the cells [8]), and blocked the pro-apoptotic effect of BzATP (Fig. 11B). Pre-treatment with random-control oligonucleotides had no effect on P2X_7_-receptor expression (Fig. 11B, insert), or on baseline apoptosis and the apoptosis induced by BzATP (Fig. 11B).

The dependence of the pro-apoptotic effect of BzATP on the expression of the P2X_7 _receptor was further demonstrated in experiments using keratinocytes obtained from P2X_7_-receptor – deficient mice. Compared to wild-type mouse keratinocytes, in both the P2X_7_-/-Pf and P2X_7_-/-GSK keratinocytes treatment with 100 μM BzATP failed to induce apoptosis (Fig. 11C).

#### Formation of P2X_7 _pores

In uterine epithelial cells [23], as well as in other types of cells [11-13], P2X_7_-receptor – dependent apoptosis involves agonist-induced acute calcium influx via P2X_7 _pores. To understand whether BzATP-induced apoptosis in mouse keratinocytes involves formation of P2X_7 _pores, experiments compared activation by BzATP of the P2X_7 _receptor (in terms of BzATP-induced increase in cytosolic calcium [23]), and the formation of P2X_7 _pores (in terms of BzATP-induced increase in the influx of ethidium bromide [23]).

**Figure 12:**
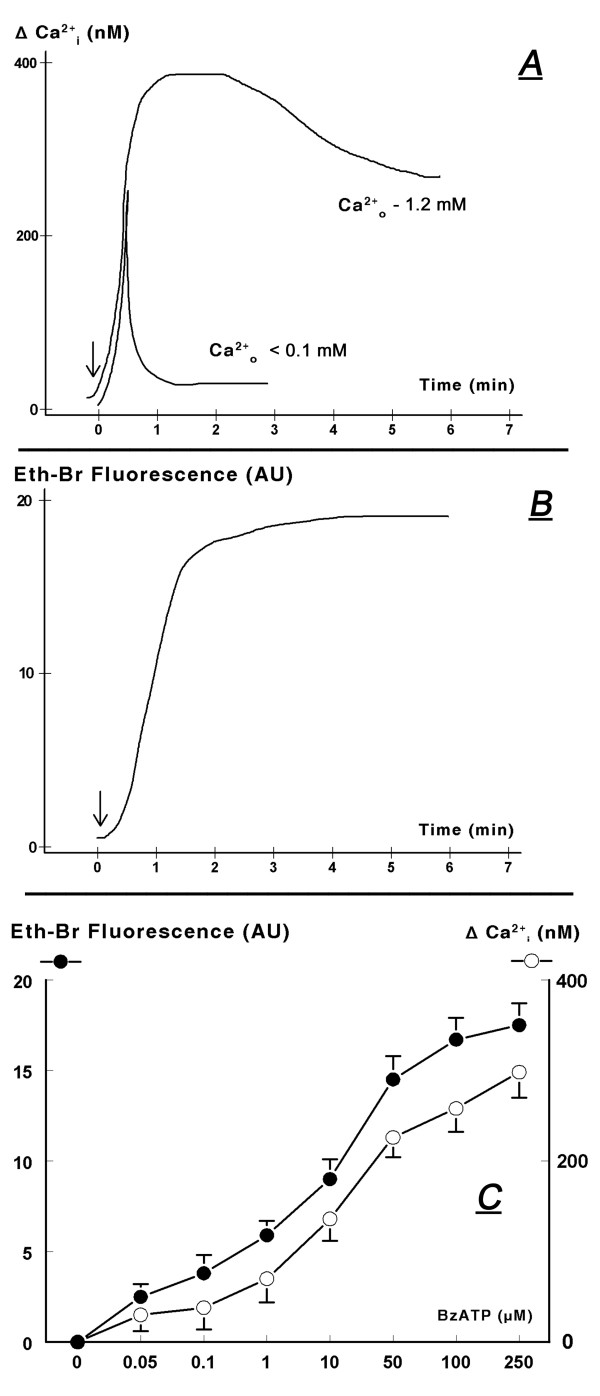
**Effects of BzATP in mouse wild-type normal keratinocytes on increases in cytosolic calcium (above baseline, ΔCa^2+^_i_), in medium containing 1.2 mM Ca^2+ ^or 1.2 mM Ca^2+ ^plus 1.2 mM EGTA (A, C); and on the influx of ethidium bromide (Eth-Br, B, C)**. Ca^2+^_o_: extracellular calcium. In **A **and **B **BzATP was added (arrows) at 100 μM. In **C **cells were treated with one of the indicated concentrations of BzATP for 8 hours. Levels of ΔCa^2+^_i _(empty circles) were determined 2 min after adding BzATP; changes in Eth-Br fluorescence (filled circles) were determined 5 min after adding BzATP. Values in **C **are means (± SD). Experiments were repeated 3 times. AU – arbitrary units.

In mouse wild-type keratinocytes treatment with 100 μM BzATP induced acute increase in cytosolic calcium, which lasted at least 6 min (Fig. 12A). In cells bathed in low calcium BzATP induced only spiked increase in cytosolic calcium, while the prolonged sustained increase in cytosolic calcium was abolished (Fig. 12A). The spiked, short-term increase in cytosolic calcium most likely represents calcium release from intracellular stores [8]. The lack of prolonged increase in cytosolic calcium in cells bathed in extracellular medium low in calcium indicates that the BzATP-induced prolonged increase in cytosolic calcium involves calcium influx.

Experiments using mouse wild-type keratinocytes also revealed that treatment with BzATP induced an acute increase in the influx of ethidium bromide (Fig. 12B) with a time-course similar to the increase in cytosolic calcium (Figs. 12A, B). Both effects had similar dose-dependence for BzATP (Fig. 12C), and they resembled the dose-dependence of apoptosis on BzATP with threshold effects at 50–100 nM and pre-maximal responses at 100–250 μM (Figs. 11A, 12C).

#### BzATP-induced increases in cytosolic calcium and influx of ethidium bromide depend on the expression of the P2X_7 _receptor

**Figure 13:**
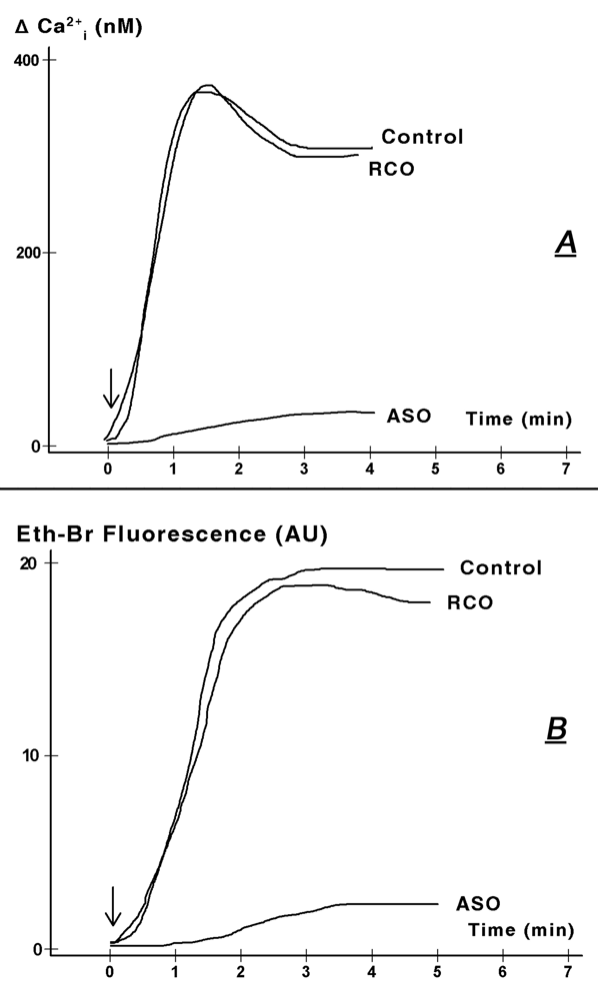
**Effects of anti-sense P2X_7 _oligonucleotides on BzATP-induced increase in cytosolic calcium (ΔCa^2+^_i_, A) and on the influx of ethidium bromide (Eth-Br, B)**. Cultured mouse wild-type normal keratinocytes were pre-treated with 100 μM anti-sense P2X_7 _oligonucleotides (ASO) or random-control P2X_7 _oligonucleotides (RCO) for 14 hours followed by 8 hours treatment with 100 μM BzATP. Control – cells treated with the vehicle of the ASO. Levels of ΔCa^2+^_i _(**A**) and of Eth-Br fluorescence (**B**) were determined as in Fig. 12. The experiments were repeated twice with similar trends.

Similar to the effects of BzATP on apoptosis, pre-treatment with the P2X_7_-receptor anti-sense oligonucleotide blocked the BzATP-induced increase in cytosolic calcium (Fig. 13A) and the BzATP-induced increase in ethidium bromide (Fig. 13B). Pre-treatment with the random-control oligonucleotides had no effect on the responses to BzATP (Fig. 13).

#### The BzATP-augmented apoptosis depends on extracellular calcium

**Figure 14:**
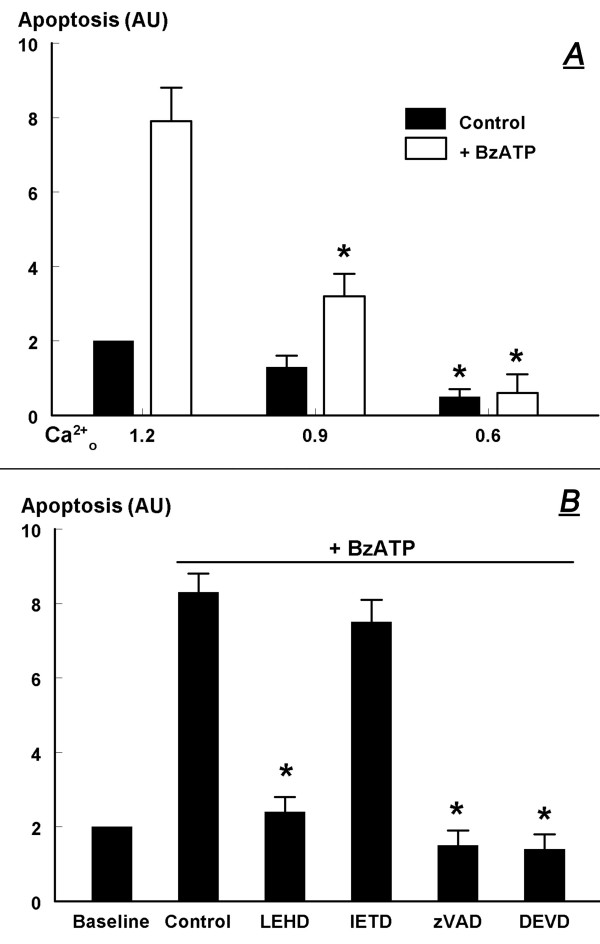
**A. Dependence of the BzATP-induced apoptosis on extracellular calcium (Ca^2+^_o_)**. Cultured mouse wild-type normal keratinocytes were shifted for 10 minutes to medium containing one of the indicated Ca^2+^_o _concentrations. Control (physiological) level of Ca^2+^_o _was 1.2 mM, and levels of Ca^2+^_o _were modulated by adding EGTA. Cells were treated with 100 μM BzATP (or the vehicle, Control), and changes in apoptosis were determined after 8 hours. **B**. Modulation of BzATP-induced apoptosis (100 μM, 8 hours) in mouse wild-type normal keratinocytes by caspase inhibitors (each added at 50 μM for 8 hours). In **A **and **B **changes in apoptosis were determined in terms of solubilized DNA. Values are means (± SD) of 3 experiments. Levels of apoptosis were normalized to an arbitrary value of 2 in non-treated cells. AU – arbitrary units. In **A**, * – p < 0.01 compared to Ca^2+^_o _1.2 mM. In **B**, * – p < 0.01 compared to control.

In mouse wild-type keratinocytes lowering extracellular calcium attenuated baseline apoptosis and blocked the BzATP-induced apoptosis in a dose-related manner (Fig. 14A).

#### The BzATP-augmented apoptosis involves caspase-9 and caspase-3

Treatment of mouse wild-type keratinocytes with the caspase-9 inhibitor LEHD-FMK blocked BzATP-induced apoptosis while the caspase-8 inhibitor IETD-FMK did not have a significant effect (Fig. 14B). The positive controls were DEVD-FMK (specific inhibitor of the terminal caspase-3) and zVAD-FMK (non-specific pan-caspase inhibitor) which similarly blocked the BzATP-induced apoptosis (Fig. 14B).

### Treatment with BzATP did not induce cell proliferation

**Figure 15:**
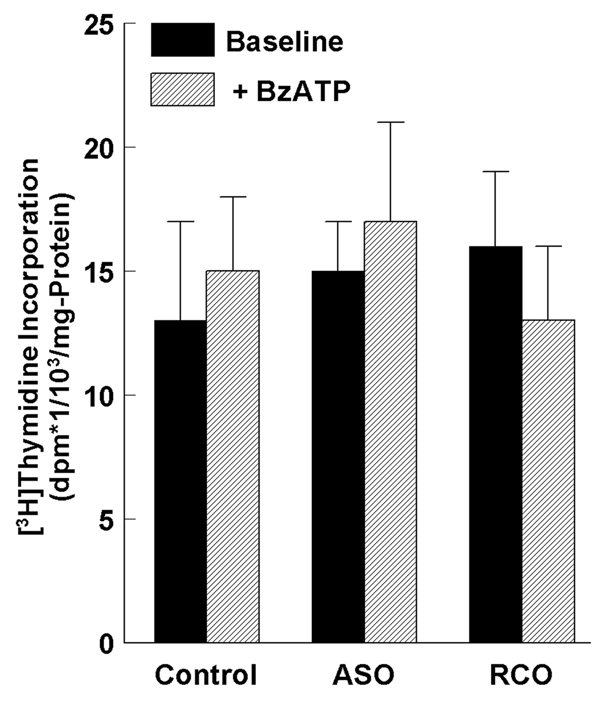
**Effects of pre-treatment with anti-sense P2X_7 _oligonucleotides (ASO) or random-control P2X_7 _oligonucleotides (RCO) (both at 100 μM for 14 hours), and of treatments with BzATP (100 μM, 8 hours) on [^3^H]thymidine incorporation in mouse wild-type normal keratinocytes (values are means ± SD, n = 4)**.

To determine if the development of the large cancerous lesions in some animals in the DMBA/TPA+BzATP group was the result of a pro-mitogenic effect of BzATP, rates of DNA synthesis (in terms of [^3^H]thymidine incorporation) in response to BzATP were measured in mouse wild-type normal keratinocytes. Pre-treatments with the P2X_7_-receptor anti-sense P2X_7 _oligonucleotides or the random-control oligonucleotides, and treatments with BzATP had no significant effect on [^3^H]thymidine incorporation (Fig. 15).

## Discussion

The main finding of the study was that pharmacological activation of P2X_7_-mediated apoptosis, by local skin application of the P2X_7_-receptor agonist BzATP, inhibited DMBA/TPA-induced formation of skin papillomas and squamous spindle-cell carcinomas. Since the main cellular effect of BzATP was augmentation of apoptosis, this discovery provides the first direct support for the hypothesis that apoptosis is an important mechanism in vivo which controls the development and progression of neoplasia. The present findings also support the hypothesis that P2X_7 _is an important physiological pro-apoptotic system in epithelia, particularly those derived from the ectoderm (skin and breast), the uro-genital sinus (bladder), and the distal paramesonephric duct (uterine cervix and endometrium) [9,38,41], and Li, Qi, Zhou, Fu, Abdul-Karim, MacLennan, and Gorodeski GI: P2X_7 _receptor expression is decreased in epithelial cancer cells of ectodermal, uro-genital sinus, and distal paramesonephric-duct origin (submitted, 2009).

Co-treatment with BzATP delayed formation of DMBA/TPA-induced papillomas, and resulted in fewer and smaller papillomas. Some papillomas regressed and involuted spontaneously, as was previously described [35], and the effect was unrelated to the treatment with BzATP. However, the majority (about two thirds) either progressed into squamous spindle-cell carcinomas or persisted as non-cancerous lesions. The latter trends depended on whether animals were co-treated with BzATP; thus, in mice co-treated with BzATP the proportion of animals with cancers at week 14 was lower than in the DMBA/TPA+BzATP group (50% versus 80%) and remained relatively stable, while in the DMBA/TPA group the proportion of animals with cancers increased steadily, reaching 100% at week 24. These data suggest that local treatment with BzATP inhibits formation of DMBA/TPA-induced skin papilloma, and it can also inhibit papilloma transformation into cancers.

BzATP had little effect on the number of cancerous lesions per animal at weeks 14–28, and on the proportion of animals with cancerous lesions > 10 mm^3 ^at weeks 14–22. In contrast, after week 23 the proportion of living animals with cancerous lesions > 10 mm^3 ^increased in the DMBA/TPA group while it had decreased in the DMBA/TPA+BzATP group. These data suggest that local treatment with BzATP exerts an inhibitory effect on the development on skin neoplasia. Interestingly, at weeks 15–24, among animals with cancerous lesions, the proportion of living animals with lesions larger than 200 mm^3 ^tended to be higher in the DMBA/TPA+BzATP group than in the DMBA/TPA group. This effect cannot be explained by augmented proliferation since BzATP did not stimulate DNA synthesis in cultured normal keratinocytes. Instead, the effect could be explained by comparing the survival curves (Fig. 6B) and the proportions of animals with smaller and larger size cancerous lesions (Figs. 5B, C). Thus, cancer-related deaths in the DMBA/TPA group were associated more often with smaller lesions while cancer-related deaths in the DMBA/TPA+BzATP group were associated with relatively larger lesions. This suggests that treatment with BzATP also prolonged the life of animals with developed cancers.

The data showed that the main targets of BzATP in the normal skin are proliferating keratinocytes of the epidermal basal/parabasal layers and hair shafts. In these P2X_7_-receptor – expressing cells BzATP augmented apoptosis without evoking inflammatory changes that potentially could have been induced by activation of the P2X_7 _receptor [11,12]. Experiments in P2X_7_-deficient normal keratinocytes and in normal keratinocytes treated with anti-sense P2X_7 _oligonucleotides showed that the P2X_7 _receptor is a necessary mediator of the pro-apoptotic effect of BzATP, suggesting that the effect of BzATP is mediated by augmentation of P2X_7_-mediated apoptosis.

Similar to the normal skin, the main targets of BzATP in papilloma tissues were P2X_7_-receptor expressing proliferating keratinocytes at the base of developing papillomas. The importance of this finding relates to the fact that in the mouse DMBA/TPA model, papillomas at risk for developing into cancer are characterized by rapidly proliferating keratinocytes in the basal and parabasal layers of the papilloma [35]. Since treatment with BzATP decreased the incidence of DMBA/TPA-induced papillomas and their transformation into cancer, it is likely that the cellular mechanism of BzATP action involved augmented apoptosis of proliferating papilloma keratinocytes bearing the potential of malignant transformation.

One of the differences between BzATP effects in the normal skin and in papilloma tissues was the lack of macroscopic effects in the former, while inhibiting the development and growth of papillomas. Thus, treatment with BzATP for 16 weeks in normal mice augmented apoptosis of proliferating keratinocytes but it did not produce thinning or ulceration of the skin, as would be expected of a potent pro-apoptotic drug. Similarly, there were no significant differences in the morphological and histological characteristics of the unaffected normal skin between animals in the DMBA/TPA+BzATP group (BzATP treatment for 30 weeks) and the DMBA/TPA group. However, in the DMBA/TPA+BzATP group the enhanced apoptosis was associated with inhibition of papilloma development. The disparity between BzATP effects in normal and papilloma tissues could be related to differences in the growth rate of the respective keratinocytes. Normal skin cells are slow growing and their overall growth rate is apparently not affected by BzATP; in contrast, in the fast growing papilloma keratinocytes BzATP-induced apoptosis slows and inhibits growth.

The data in normal mice also showed that local treatment with BzATP had no adverse systemic effects, suggesting a relatively safe profile for the drug when applied locally on the skin. These data indicate that BzATP is absorbed from the skin into the basal/parabasal epidermal regions and hair shafts. The data also suggest that the predominant effect of BzATP is induction of apoptosis at the site of application, targeting rapidly growing proliferating keratinocytes.

In contrast to papillomas, the expression level of P2X_7 _receptors in DMBA/TPA-induced cancer cells was low, as was evident by three assays: in-situ immunoreactivity, Western blots, and qPCR. These findings are similar to those reported in non-melanoma skin cancer cells [30] and in uterine, bladder and breast epithelial cancers [9,38,41], and Li, Qi, Zhou, Fu, Abdul-Karim, MacLennan, and Gorodeski GI: P2X_7 _receptor expression is decreased in epithelial cancer cells of ectodermal, uro-genital sinus, and distal paramesonephric-duct origin (submitted, 2009). The findings suggest that the rapid proliferation of cancer cells could be in part due to the low expression of the P2X_7 _receptor and to attenuated P2X_7_-mediated apoptosis. Treatment with BzATP augmented apoptosis even in cancer cells expressing low levels of the receptor, but the effect was smaller than in normal or papilloma cells. The significance of this effect is at present unclear although it could have modified the biological behavior of the cancers and have contributed to the prolongation of life in the affected animals, as was discussed above.

Until recently little was known about the mechanisms of P2X_7_-receptor – apoptosis in the skin, and one of the objectives of the present study was to begin to understand the signaling pathways and molecular mechanisms that are involved in BzATP action in keratinocytes. The data suggest that, similar to uterine epithelial cells [8,18], the P2X_7_-receptor – apoptosis in keratinocytes depends on enhanced calcium influx via P2X_7 _pores, and is mediated by the caspase-9 – mitochondrial pathway. The following experimental findings in the present study support this hypothesis: (a) Treatment with BzATP induced formation of pores and enhanced calcium influx; (b) the BzATP-induced apoptosis, pore formation and the augmented and prolonged calcium influx were critically dependent on the expression of the P2X_7 _receptor; (c) the BzATP-induced apoptosis, pore formation and the augmented calcium influx had similar dose-dependence on BzATP; (d) the BzATP-induced pore formation and the augmented calcium influx began shortly (30–60 seconds) after adding BzATP. In contrast, the BzATP-induced apoptosis required hours of treatment with BzATP, commensurate with a gene-mediated effect; (e) the BzATP-induced apoptosis depended on the presence of extracellular calcium at a physiological concentration of 1.2 mM, and on calcium influx; (f) the BzATP-induced apoptosis could be blocked by co-treatment with inhibitors of caspase-9 and caspase-3, but not of caspase-8. Since caspase-3 is a terminal step in the caspase cascade [5,6], a possible interpretation of the present results is that P2X_7_-receptor – apoptosis is mediated by the caspase-9 (mitochondrial) pathway. Collectively the data in mouse keratinocytes suggest that BzATP-dependent activation of the P2X_7 _receptor involves formation of pores in the plasma membrane, and that facilitated uncontrolled influx of Ca^2+ ^via the P2X_7 _pores stimulates apoptosis by the mitochondrial – caspase-9 pathway.

P2X_7 _pores are believed to be formed of channels composed of pannexins [42,43] and ectodomains of the P2X_7 _molecule [23,42]. However, the ability of agonists to induce apoptosis via the P2X_7 _pore mechanism is determined primarily by the cellular expression of the P2X_7 _receptor [13,23]. The present study showed that papilloma keratinocytes express the P2X_7 _receptor; therefore, the high expression levels of the receptor in papilloma cells and the significant apoptotic effects in response to BzATP could explain the inhibitory effect of BzATP on papilloma development. In contrast, the lesser effect of BzATP in skin cancer cells could be explained by the low expression level of the P2X_7 _receptor in the cancer cells.

At present little is known whether the neoplastic transformation induces lesser expression of the P2X_7_-receptor, or whether the neoplastic transformation is triggered preferentially in cells expressing low levels of the receptor. The former possibility is supported by data in endometrial and bladder cells where low expression of the P2X_7 _receptor was found already in pre-cancerous and early cancerous cells but not in hyperplastic benign cells [38]. Accordingly, the carcinogenic process could have induced lesser expression of the P2X_7 _already at early stages of cancer development. On the other hand the possibility that the neoplastic transformation is triggered preferentially in cells expressing low levels of the receptor is supported by data as well, and it could be more fundamental to the understanding of epithelial-cell carcinogenesis. Thus, in uterine cervical epithelia low expression of the P2X_7 _receptor was found already in dysplastic cells [9]. Since only a small fraction of cervical dysplasia cases progresses to cancer [44-46], it is possible that low expression of the P2X_7 _receptor in the cervix precedes the neoplastic transformation. Accordingly, abrogation of P2X_7_-mediated apoptosis could be responsible for the preservation of genetically aberrant cells that are susceptible to carcinogenic stimuli, favoring neoplastic transformation [47].

The present data showed only partial inhibition (by about 50%) of papilloma and cancer formation in BzATP-treated mice. The experiments used the relatively low dose of 1 μg/cm^2 ^BzATP, based on the 100 μM concentration used in experiments with cultured cells. The study was not designed to test higher doses and different frequencies of drug administration, and it is possible that higher doses and/or more frequent applications could produce greater inhibition papillomas and cancers. Additional studies are needed to test this possibility.

In addition to improving our understanding of the biogenesis of skin cancers and possibly other types of epithelial cancers where the P2X_7 _controls cell growth, the present results provide a basis for continued research of novel chemotherapeutic growth-preventive modalities through regulation of apoptosis. The rationale is that epithelial cancers usually develop from premalignant lesions, e.g. papilloma, and the cancer risk of premalignant epithelial lesions may vary from 0.1% to 20% [48-50]. The present results in the mouse model showed that local treatment with P2X_7_-receptor agonists could inhibit the development of papillomas and inhibit the transformation of papillomas into cancers. BzATP appears to be a candidate chemotherapeutic growth-preventive drug for skin papillomas, with an apparent low risk profile of adverse events when administered locally on the skin. However, more studies are needed to test whether BzATP could be used in humans.

## Conclusion

• P2X_7_-dependent apoptosis is an important mechanism that controls the development and progression of epidermal neoplasia in the mouse.

• P2X_7_-dependent apoptosis in keratinocytes is mediated by calcium influx via P2X_7 _pores, and involves the caspase-9 (mitochondrial) pathway.

• The diminished pro-apoptotic effect of BzATP in mouse cancer keratinocytes is possibly the result of low expression of the P2X_7 _receptor.

• Activation of P2X_7_-dependent apoptosis, e.g. with BzATP could be a novel chemotherapeutic growth-preventive modality for papillomas and epithelial cancers in vivo.

## Competing interests

CytoCore Inc. funded a small part of the study but it has no financial interest in the study. Dr. Gorodeski was paid consultant to CytoCore Inc. and he holds restricted stocks of CytoCore. The ties between Dr. Gorodeski and CytoCore were severed in March 2008 and Dr. Gorodeski has no financial interest in the study. None of the other authors had any ties with CytoCore. Neither Dr. Gorodeski, nor any of the other authors have other financial or non-financial competing interests. University Hospital CASE Medical Center and Case Western Reserve University have a financial interest in the study

## Authors' contributions

WF carried out the animals' experiments. TM supervised the animals' experiments. XQ carried out the immunostaining assays. LL assisted with the animals' experiments. LZ and XL carried out the cell culture assays. BCW participated in its design of the animals experiments. HG participated in its design of the study and carried out the data analysis. FWAK evaluated the pathology results. GIG conceived the study; participated in its design and coordination; and drafted the manuscript. All authors read and approved the final manuscript.

## Pre-publication history

The pre-publication history for this paper can be accessed here:

http://www.biomedcentral.com/1471-2407/9/114/prepub
